# Microfluidic Screening Reveals Heparan Sulfate Enhances Human Mesenchymal Stem Cell Growth by Modulating Fibroblast Growth Factor‐2 Transport

**DOI:** 10.1002/sctm.16-0343

**Published:** 2017-02-16

**Authors:** Drew M. Titmarsh, Clarissa L.L. Tan, Nick R. Glass, Victor Nurcombe, Justin J. Cooper‐White, Simon M. Cool

**Affiliations:** ^1^ Institute of Medical Biology, Agency for Science Technology and Research (A*STAR)Singapore; ^2^ Australian Institute for Bioengineering & Nanotechnology; ^3^ School of Chemical Engineering, The University of Queensland, St. LuciaQueenslandAustralia; ^4^ Lee Kong Chian School of Medicine, Nanyang Technological University–Imperial College LondonSingapore; ^5^ Biomedical ManufacturingManufacturing Flagship, CSIROClaytonVictoriaAustralia; ^6^ Department of Orthopaedic SurgeryYong Loo Lin School of Medicine, National University of SingaporeSingapore

**Keywords:** Microfluidics, Heparan sulfate, Fibroblast growth factor 2, Mesenchymal stromal cells

## Abstract

Cost‐effective expansion of human mesenchymal stem/stromal cells (hMSCs) remains a key challenge for their widespread clinical deployment. Fibroblast growth factor‐2 (FGF‐2) is a key hMSC mitogen often supplemented to increase hMSC growth rates. However, hMSCs also produce endogenous FGF‐2, which critically interacts with cell surface heparan sulfate (HS). We assessed the interplay of FGF‐2 with a heparan sulfate variant (HS8) engineered to bind FGF‐2 and potentiate its activity. Bone marrow‐derived hMSCs were screened in perfused microbioreactor arrays (MBAs), showing that HS8 (50 μg/ml) increased hMSC proliferation and cell number after 3 days, with an effect equivalent to FGF‐2 (50 ng/ml). In combination, the effects of HS8 and FGF‐2 were additive. Differential cell responses, from upstream to downstream culture chambers under constant flow of media in the MBA, provided insights into modulation of FGF‐2 transport by HS8. HS8 treatment induced proliferation mainly in the downstream chambers, suggesting a requirement for endogenous FGF‐2 accumulation, whereas responses to FGF‐2 occurred primarily in the upstream chambers. Adding HS8 along with FGF‐2, however, maximized the range of FGF‐2 effectiveness. Measurements of FGF‐2 in static cultures then revealed that this was because HS8 caused increased endogenous FGF‐2 production and liberated FGF‐2 from the cell surface into the supernatant. HS8 also sustained levels of supplemented FGF‐2 available over 3 days. These results suggest HS8 enhances hMSC proliferation and expansion by leveraging endogenous FGF‐2 production and maximizing the effect of supplemented FGF‐2. This is an exciting strategy for cost‐effective expansion of hMSCs. Stem Cells Translational Medicine *2017;6:1178–1190*

**Significance Statement**

Clinical use of stem cells will require effective methods to expand stem cell numbers. This work used a purpose‐engineered carbohydrate to target a key growth factor pathway that facilitates stem cell expansion, which is potentially a cost‐effective alternative to current expansion techniques. To understand the interplay of the carbohydrate with the growth factor, and its effects on stem cells, a microbioreactor array—a “lab‐on‐a‐chip” system—was used to assess many different conditions in parallel, streamlining the process of optimizing the culture conditions and understanding the mechanism of action of the carbohydrate.

## Introduction

Human mesenchymal stem/stromal cells (hMSCs) 1 are currently undergoing both preclinical and clinical development for a range of indications due to their multi‐lineage differentiation capacity, and propensity for secretion of trophic and immunomodulatory factors 2. The large numbers of hMSCs required for clinical‐scale dosing are typically obtained by ex vivo expansion of isolated primary cells from bone marrow aspirates, amongst other sources. Human MSCs generally display reduced proliferative and differentiation capacity following extended ex vivo expansion 3. Therefore, culture strategies that achieve robust cell number expansion whilst maintaining sufficient therapeutic potency must be developed, and further optimized to ensure economic viability of hMSC bioprocessing at clinical scale. As a way to rapidly test and optimize hMSC expansion strategies at a small scale, microfluidic cell culture tools are available that allow for multiplexed generation and testing of cell culture conditions 4. In this work, we use the versatility of microfluidic cell culture arrays—microbioreactor arrays (MBAs) 5, 6—to rapidly screen the impact of combinations of factors on hMSCs.

Human MSCs are known to produce a plethora of growth factors at varying levels under normal expansion conditions 7, 8. Given the availability of these endogenous factors, strategies that successfully exploit them for cell expansion would be economically attractive alternatives to supplementing medium with large amounts of expensive recombinant factors, particularly at industrial scales. Notably, these endogenously‐produced factors include high levels of fibroblast growth factor‐2 (FGF‐2) 8, which is known to be a potent mitogen for hMSCs 9. FGFs potentiate a signaling cascade through FGF receptors (FGFRs) 1‐4 10, and in hMSCs, cell cycle progression driven by FGF‐2 signaling through FGFR1 has been identified as a rate‐limiting step for hMSC self‐renewal 11. Expression of endogenous FGF‐2 at higher levels in adipose‐derived versus bone marrow‐derived hMSCs was highlighted as a factor leading to increased growth rates in the adipose‐derived cells 12. Confirmation of both FGF‐2 and FGFR1 expression in adipose‐derived hMSCs revealed the presence of an autocrine feedback loop, with fast‐cycling cells also having higher levels of cell surface FGF‐2 than slow‐cycling cells 13. In hMSCs, exogenous FGF‐2 treatment resulted in early preferential expansion of progenitors with shorter telomeres and enhanced proliferative and differentiation potential 9, 14, 15. Likewise, in mouse MSCs, exogenous FGF‐2 selectively expanded subpopulations containing immunomodulatory and anti‐inflammatory capacity 16, suggesting this signaling axis may be important for therapeutically desirable cells within the MSC population. However, extended treatment with supplemented FGF‐2 over multiple passages has also been suggested to impact the differentiation potential of the cells 17, potentially an indicator of compromised potency.

Endogenous FGF‐2 secretion is dependent on native heparan sulfate (HS) chains proximal to the cell membrane 18, which then bind the exported FGF‐2. This anchoring of the factor to the cell membrane through association with heparan sulfate proteoglycans (HSPGs) is expected to constrain the effective diffusion of FGF‐2 to the cell surface, increasing the ease of FGF association with its receptor on the same exporting cell, thus promoting an autocrine signaling mechanism. It also limits paracrine diffusive transport of exported FGF‐2 through the bulk medium, but transfer can occur between physically touching cells 18—juxtacrine signaling. This has important repercussions in a heterogeneous cell population such as hMSCs—non‐FGF‐2‐producing cells may not receive FGF‐2 if not in physical contact with a producer cell. Conversely, the FGF‐2/FGFR1 axis may not be targeted across the whole population if an appropriate HS chain acting as an FGF‐2 co‐receptor is not present. The proportions and overlap between the FGF‐2‐producing and FGFR1‐displaying cell populations, as well as the distribution of FGF‐2‐binding HS chains, is not well understood in hMSCs.

Exogenously supplemented FGF‐2, on the other hand, is known to be unstable due to thermal aggregation and thermal and enzymatic degradation. FGF‐2 bioactivity decays and it loses the ability to cause phosphorylation of extracellular signal‐regulated kinase (ERK) after 24‐hours incubation in medium at 37°C, yet this can be rescued by binding of heparin to FGF‐2 protein 19, thus researchers commonly use heparin to stabilize FGF proteins 20. Stabilization of FGF‐2 by binding of heparin or free HS chains (not bound to cell surface HSPGs) has been shown to increase its radius of diffusion on a monolayer of cells 21. This was thought to occur by partitioning FGF‐2 into the bulk medium, presumably by competing with endogenous, cell surface HS chains for FGF‐2 binding. Together, these factors give rise to an unfavorable situation whereby the activity and availability of supplemented exogenous FGF‐2 may be adversely limited unless it is appropriately complexed with heparin or HS, and the endogenously produced FGF‐2 is limited to autocrine or juxtacrine transport. Thus, FGF‐2 availability might be a limiting factor in ex vivo hMSC expansion.

We have previously shown that supplementation of hMSC cultures with the generic hyper‐sulfated glycosaminoglycan (GAG) heparin, results in adverse changes in the biological properties of the cells 22. However, HS preparations can selectively bind FGF‐2 and potentiate its signaling 23, improving growth and potency of hMSCs 24. This highlights the importance of using a particular HS variant that targets a specific factor like FGF‐2, rather than a nonselective GAG such as heparin, and suggests that HS‐based strategies might be attractive to enhance FGF‐2 availability and activity. In this work, we utilize a HS variant (HS8) engineered to bind FGF‐2 with high affinity and potentiate its activity, the characterization of which we recently reported 25. HS8 comprises a more targeted population of HS chains that is enriched for FGF‐2 binding affinity compared to the crude HS starting material.

To explore the utility of HS8 for hMSC expansion, we used MBAs to screen combinations of FGF‐2 and HS8 on hMSCs and dissected the interactions between HS8 and endogenous and exogenous FGF‐2. The MBA platform is a microfluidic cell culture‐screening array providing both combinatorial mixing of factors, and continual perfusion of culture medium through serially connected culture chambers. In this device, progressive accumulation of cell secretions from upstream to downstream chambers introduces paracrine factor effects, enabling not only a rapid manner by which to search for preferential combinations of exogenous factors, but potentially the elucidation of difficult to detect paracrine‐dependent cell responses. Deployment of the MBA is intended to save on time, biological materials, and manual errors, while increasing the landscape of cell culture environments that can be rapidly generated and compared side‐by‐side, with the end goal of utilizing it as a rapid development and QC tool for cell therapies. With this approach, we gain insight into the interplay between FGF‐2 and HS8 and the effects on hMSC growth.

## Materials and Methods

All fine chemicals were obtained from Sigma Aldrich (Singapore, http://www.sigmaaldrich.com), and all cell culture and detection reagents from Life Technologies (Singapore, http://www.thermofisher.com), unless otherwise mentioned. FGF‐2 was purchased from R&D Systems (Cat# 233‐FB; Minneapolis, MN; http://www.rndsystems.com). The engineering of HS8, including isolation, biochemical characterization, and evaluation of biological activity, including on hMSCs, was recently published by our group 25.

### Microbioreactor Array Fabrication and Validation

MBAs 5, 6, 26 are microfluidic cell culture devices incorporating a cell culture array of 270 chambers (27 columns of 10 chambers linked in series by microfluidic channels), with diameter 1.63 mm and height 100 μm. Connected to the cell culture array are fluidic channels for combinatorial factor mixing, and inlet and outlet structures for various operations (cell seeding, cell culture, cell analysis). This arrangement allows cells to be seeded and attach in the cell culture array, and then be exposed to continuous flow of a combinatorial mix of test factors, then finally to be assayed with various fluorescent tags and imaged for analysis. MBAs were fabricated to 100 μm feature height, and validated by dye loading and fluorimetric concentration quantification, similar to previous work 5, 6. For dye loading, 0.1% wt/vol Ponceau S solution (Bio‐Rad; Singapore; http://www.bio-rad.com; 672.64 Da) was perfused through individual factor channels, with all other inlets containing MilliQ water, at 1.2 ml/hour total flow rate. For concentration quantification, heparin was selected as a model biomolecule for HS due to its structural and charge similarities. Heparin was conjugated to Alexa Fluor 488 dye, according to previously described methods 27. Alexa Fluor 488‐labeled heparin (10 μg/ml in PBS) was perfused through individual factor channels independently, with all other inlets containing PBS, at 300 μl/hour total flow rate. Chambers were imaged (see below), integrated fluorescence intensities were measured using FIJI software (http://fiji.sc), then the intensities had background subtracted and were normalized to the minimum and maximum fluorescence values.

### Cell Culture

Normal hMSCs were obtained from Lonza (Poietics, PT‐2501; Donor A; healthy male, 27 years; lot number 0000318006; Singapore, http://www.lonza.com) at passage 2. Alternatively, bone marrow mononuclear cells were obtained from Lonza (2M‐125C), and then hMSCs isolated by plastic adherence and culture according to standard methods. This donor (Donor B; healthy male, 29 years, lot number 090016B) was characterized for its immunophenotype (Supporting Information Fig. 1) and multi‐lineage differentiation potential (Supporting Information Fig. 2). A third donor (Donor C; healthy male, 24 years, lot number 090166D) was isolated in the same manner. Human MSCs were expanded in maintenance medium consisting of low‐glucose Dulbecco's Modified Eagle Medium (DMEM‐LG) (HyClone; Singapore; http://www.gelifesciences.com), 10% vol/vol fetal calf serum (HyClone), and 4 mM l‐glutamine. Cells were plated at 5 × 10^3^ cells/cm^2^ in 150 mm culture dishes and grown in a 5% CO_2_ humidified atmosphere at 37°C. Medium was exchanged every 3 days and cells were passaged at ∼90% confluence. Cells were used at passage numbers as indicated. All media were supplemented with 1% vol/vol penicillin/streptomycin.

### Human MSC Multi‐lineage Differentiation

#### Adipogenic Differentiation

Human MSCs were plated in triplicate at 1.8 × 10^4^ cells/cm^2^ in 12‐well plates and cultured for 3 days. When confluent, medium was replaced with Adipogenic Induction Medium (AIM) comprising maintenance medium supplemented with 1 µM dexamethasone, 10 µM insulin, 20 µM indomethacin, and 115 µg/ml 3‐isobutyl‐1‐methylxanthine. Cells were maintained in AIM for 14 days, with AIM prepared fresh and replaced every 3–4 days. Undifferentiated cells used as controls (for each lineage) were kept in maintenance media for 14 days with replacement every 3–4 days.

#### Osteogenic Differentiation

Human MSCs were plated in triplicate at 3 × 10^3^ cells/cm^2^ in 12‐well plates and allowed to attach overnight. Then, medium was replaced with Osteogenic Induction Medium (OIM) comprising of maintenance medium supplemented with 10 nM dexamethasone, 25 µg/ml l‐ascorbate‐2‐phosphate and 10 mM glycerol‐2‐phosphate. Cells were maintained in OIM for 14 days and medium was changed every 3–4 days.

#### Chondrogenic Differentiation

Human MSCs (2.5 × 10^4^ cells) were pelleted in triplicate in Chondrogenic Induction Media (CIM; Lonza) supplemented with 10 ng/ml TGF‐β3 (R&D Systems) in 15 ml centrifugation tubes. Cells were maintained in CIM for 14 days and medium was changed every 3–4 days.

### Flow Cytometry

TrypLE‐dissociated hMSC suspensions were stained (as described previously 8) with PE‐ or FITC‐conjugated antibodies against human CD105, CD73, CD90, CD45, CD34, CD49a, CD29, EGF‐R, IGF‐IRα, NGF‐R, PDGFRα, PDGFRβ, CD11b, HLA‐DR, CD19, CD14, CD106, CD146, SSEA‐4, and STRO‐1, or the mouse isotype‐matched controls IgG1κ, IgG2аκ, IgG2bκ, IgM, and IgG3, and analyzed on a BD FACSArray Instrument (BD Biosciences; San Jose, CA; https://www.bdbiosciences.com) and with FlowJo software v7.6.5 (FlowJo; https://www.flowjo.com/). Gates were set for a false‐positive rate of <2% based on the respective isotype control. For each sample, >10,000 events were acquired. All antibodies were purchased from BD Biosciences, except STRO‐1 (generous gift of Prof. Stan Gronthos, University of Adelaide, Australia).

### Reverse Transcriptase‐Quantitative PCR Analysis

Gene expression was measured in triplicate cultures using reverse transcription and TaqMan hydrolysis probes. Total RNA from adipogenic and osteogenic differentiated and undifferentiated cells was isolated using a Nucleospin RNA extraction kit (Macherey‐Nagel; Bethlehem, PA; http://www.mn-net.com/) following manufacturer's instructions. Total RNA from chondrogenic differentiated and undifferentiated cells was isolated using TRIzol Reagent following manufacturer's instructions. The quality and quantity of total RNA was determined by a NanoDrop spectrophotometer (Thermo Fisher Scientific). Total RNA (1 μg) was reverse‐transcribed into cDNA using a SuperScript VILO cDNA synthesis kit and an ABI Veriti 96‐well thermal cycler (Life Technologies). Expression levels of target genes were determined using 40 ng of each cDNA sample assayed in triplicate and amplified with a QuantStudio 6 Flex Real‐Time PCR system. The probes for *ALPL* (liver/bone/kidney isoenzyme), *IBSP* (also known as bone sialoprotein II), *PPARG*, *CEBPA*, *SOX9*, *COL2A1*, *ACAN,* and reference gene *ACTB* (beta‐actin) were purchased pre‐designed (probe information, Supporting Information Table 1). Expression levels were reported in Relative Expression Units (REU) normalized to *ACTB*.

### Microbioreactor Array Screening of hMSCs

The MBA experimental workflow consists of: MBA preparation and cell seeding and attachment, cell culture, and cell endpoint analysis. MBAs were first autoclaved then vacuum filled with sterile PBS. Human MSCs were detached with TrypLE, washed, and resuspended in fresh medium at 1 × 10^6^ cells/ml. Then ∼500 μl of this suspension was injected into MBAs using the cell injection ports, with other ports plugged. The cell seeding distribution between chambers in the MBA has been quantified previously to have a coefficient of variation of ∼5% for hMSCs 6. Human MSCs were allowed to attach for 8 hours in an incubator before medium perfusion was initiated, or for 24 hours with 6‐hourly exchange of medium (as indicated). Media were then perfused at 36 μl/hour total flow rate using a syringe pump (NE‐1200, New Era Pump Systems; Farmingdale, NY; http://www.syringepump.com/) situated outside the incubator, driving 1 ml syringes (BD) connected through polyethylene tubing (Intramedic PE50, BD) and stainless steel fittings to the MBA housed in a petri dish inside the incubator.

Because serum contains heparin‐binding compounds, the sequential mixing of factors in the MBA was exploited to firstly pre‐complex FGF‐2 with HS8 in the absence of serum. These factors were diluted in minimal medium (DMEM‐LG +4 mM l‐glutamine +1% penicillin/streptomycin) with 0.1% bovine serum albumin (BSA) as a carrier protein to prevent adsorption losses in tubing and fittings. Serum was then introduced uniformly in the third factor channel. Final concentrations are as marked.

At the experimental endpoint, MBAs were removed from the incubator and submerged in a bath of ice‐cold PBS. Fixation and immunostaining were then performed using the syringe pump setup to exchange staining solutions. In each case a minimum volume of 300 μl was delivered to ensure complete fluid replacement within the chip. Antibodies used were: Ki67 (1:200, Cell Signalling Technologies; Danvers, MA; https://www.cellsignal.com/) and CD90‐PE (1:20, BD). Actin and nuclei were optionally counterstained with rhodamine‐phalloidin (1:500) and Hoechst 33342 (2 μg/ml), respectively.

### Imaging

Immunostained MBAs were imaged with an Olympus IX83 inverted fluorescence microscope, with an automated stage, CoolSNAP HQ2 CCD camera (Photometrics; Tucson, AZ; http://www.photometrics.com/), and MetaMorph control and acquisition software (Molecular Devices; Sunnyvale, CA; https://www.moleculardevices.com). The ‘Scan Slide’ application in MetaMorph was used to acquire the full cell culture array section (∼63 × 23 mm), with a ×4 objective, 2 × 2 camera binning, and in multiple fluorescence channels.

### Image Processing

MBA image tiles were stitched into full‐resolution images using MetaMorph, and then these images were registered and sliced into images of individual MBA chambers using a custom MATLAB script (MathWorks; Natick, MA; https://www.mathworks.com/). Image cytometry processing was performed with CellProfiler software (version 2.1, Broad Institute; Cambridge, MA; www.cellprofiler.org) 28, 29. The processing pipeline firstly identified nuclei by segmentation based on Hoechst staining, and then identified Ki67^+^ nuclei using segmentation based on Ki67 staining. Projected cytoplasmic areas were identified using CD90 staining, with identified nuclei used as the seed structures for shape propagation, and then object measurements were exported.

### FGF‐2 Enzyme‐Linked Immuno‐Sorbent Assay

Human MSCs were plated in triplicate at 1 x 10^4^ cells/cm^2^ in 24‐well plates and allowed to attach for 24 hours. Following this cells were washed with PBS and then treated with medium alone, medium + 25 ng/ml FGF‐2, medium + 25 μg/ml HS8, or medium containing both, for 1 or 3 days. Supplements were allowed to mix on ice for 15 minutes in the absence of serum, before supplementation. Following 1‐ and 3‐days culture, cell culture supernatants were recovered and frozen. Cell layers were then treated with 2 M NaCl in 20 mM HEPES solution on an orbital shaker for 10 minutes, and then the solution was recovered and frozen at −80°C. Cell surface and supernatant fractions were later thawed and promptly assayed using a human FGF‐2 Quantikine enzyme‐linked immuno‐sorbent assay (ELISA) kit (R&D Systems) according to the manufacturer's instructions.

### Data Visualization and Statistical Analysis

Heat maps were generated with Excel (Microsoft; Redmond, WA; https://www.microsoft.com), and color‐scaled with white point at the mean. MINITAB 17 software (Minitab Inc.; State College, PA; http://www.minitab.com) was used perform factorial analyses. Prism 5 (GraphPad; La Jolla, CA; https://www.graphpad.com/) was used for comparisons by analysis of variance (ANOVA) with post hoc tests, as labeled. For factorial analyses, column means (mean of 10 serial chambers) were used, with replicates coming from independent runs from multiple donors. To compare multiple MBA runs, column mean data from each run were standard normalized to arrive at a *Z*‐score (representing standard deviations away from the global mean for that run) by: $Z=\left(x-\mu \right)/\sigma$, where *Z* is the standard normalized *Z*‐score for data point *x*, and *μ* and *σ* are the mean and standard deviation of all data points for that run, respectively.

## Results

### MBA Performance Validation With Sulfated GAG Macromolecules

**Figure 1:**
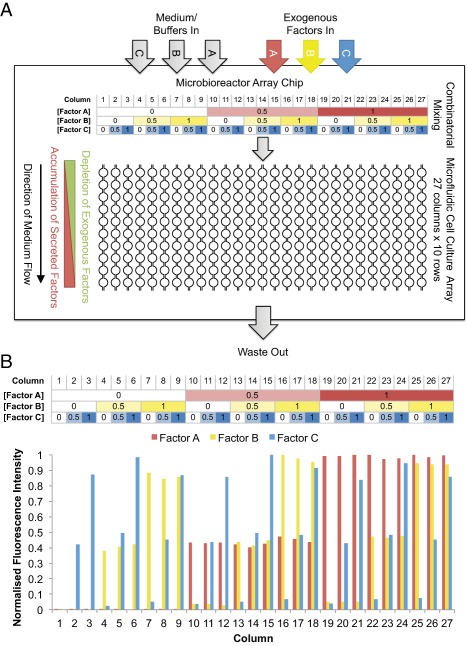
Microbioreactor arrays (MBA) design schematic and concentration validation. **(A)**: Schematic of MBA functions. **(B)**: Upper panel shows design normalized concentrations of factors in each column of the MBA. Corresponding lower panel shows normalized concentrations of Alexa Fluor 488‐labeled heparin in each column of the MBA.

The MBA 5, 6 performs two main functions (Fig. 1A). First, factors and buffers are perfused into the chip such that 3 concentrations of each of the 3 factors are generated, which are then are combinatorially mixed into 27 distinct compositions. Second, the 27 media are perfused continuously through a cell culture array of 10 serial culture chambers for each distinct composition, before exiting at a common waste outlet (Fig. 1A). We firstly confirmed that the MBA partitions factors as designed (Fig. 1A) using Ponceau S dye (Supporting Information Fig. 3). We then verified the ability of the MBA to diffusively mix sulfated GAG macromolecules (i.e., heparin and HS) to completion, by perfusing Alexa Fluor 488‐labeled heparin and measuring the lateral fluorescence profiles in serial channel segments in diffusive mixing channels (Supporting Information Fig. 4A). This confirmed that, as a model GAG macromolecule, Alexa Fluor 488‐labeled heparin could be mixed to completion by the diffusive mixing regime in the device. Fill volumes for each of the factor channels were also estimated by perfusing Alexa Fluor 488‐labeled heparin and tracking fluorescence levels over time. Fill volumes for all factor channels were within 300 μl (Supporting Information Fig. 4B). We then measured by fluorescence microscopy the relative concentration levels generated in each column of the MBA, when Alexa Fluor 488‐labeled heparin was perfused through each of the three factor channels (A, B, and C) independently and sequentially. This confirmed that the design concentrations levels were accurately generated (Fig. 1B). Residual dye detection in zero‐concentration conditions of Factor B and Factor C is due to small amounts of adsorbed Alexa Fluor 488‐labeled heparin from the previous factor channel. We do not expect significant losses to PDMS absorption 30 since as a negatively charged, hydrophilic macromolecule, HS should not be absorbed appreciably, similar to mannitol 31. Further, we have shown that labile proteins such as FGF‐2 and TGF‐β1 are delivered and active within MBAs 26, 32. These measurements collectively confirmed the MBA platform was functioning as desired for use with sulfated GAGs.

### MBA Combinatorial Screening to Map the Effects of FGF‐2, HS8, and SU5402 on hMSCs

**Figure 2:**
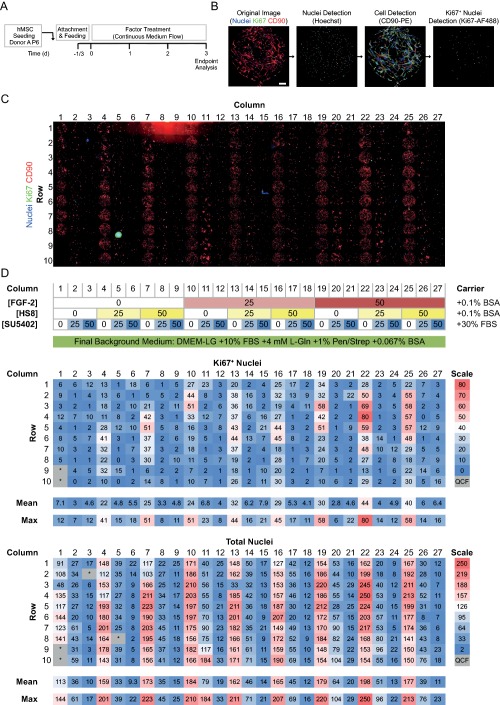
Microbioreactor arrays (MBA) combinatorial screening of FGF‐2, HS8, and SU5402 on hMSCs. **(A)**: Experimental time course. **(B)**: Steps in image processing pipeline to identify total nuclei, cell membrane area, and Ki67^+^ nuclei. Scale bar, 200 µm. **(C)**: Montaged fluorescence microscopy images of all 270 chambers in the MBA. **(D)**: Top panel: compositions of media in each column of the MBA (FGF‐2, ng/ml; HS8, µg/ml; SU5402, µM). Lower panels: Heat maps of absolute numbers of Ki67^+^ nuclei, and total nuclei for each chamber in the MBA, corresponding to above compositions. Medium flow was from top (Row 1) to bottom (Row 10) down a column. Mean and maximum measurements for each column are given below. Abbreviations: BSA, bovine serum albumin; DMEM‐LG, low‐glucose Dulbecco's Modified Eagle Medium; FBS, fetal bovine serum; FGF‐2, fibroblast growth factor‐2; HS8, heparan sulfate variant; hMSCs, human mesenchymal stem/stromal cells; QCF: data flagged for quality control issue during image processing.

To map the effects of combinations of FGF‐2 and HS8, hMSCs were seeded into MBAs and screened for 3 days (for Donor A; Fig. 2A), under the combinatorial panel of FGF‐2 (0, 25, and 50 ng/ml), HS8 (0, 25, and 50 µg/ml) and the FGFR1 receptor tyrosine kinase inhibitor SU5402 (0, 25, and 50 µM) (shown in Fig. 2D). At the endpoint, the entire MBA was immunostained for Ki67 and CD90, and counterstained for nuclei, then imaged. We then used image cytometry to enumerate absolute numbers of individual nuclei (Hoechst 33342 staining), Ki67^+^ nuclei (Ki67 staining), and to detect the cell membrane (CD90 staining) (Fig. 2B). Distinct response patterns were seen to result from the various media applied to the 27 columns of culture chambers (Fig. 2C). The number of Ki67^+^ cells per chamber (Ki67^+^ nuclei), and the total number of nuclei per chamber (total nuclei) were used as readouts of the cell proliferative and expansion responses to factor treatment. These metrics were arranged as heat maps showing absolute values for each individual chamber in the MBA, versus the combinatorial panel of factors applied to each column in the MBA (Fig. 2D). There was a strong positive correlation (Pearson's *r* = .765; *p* < .001) between the two metrics, showing chambers with the most proliferating cells (Ki67^+^ nuclei) also had higher cell numbers after the 3‐day culture period. Distinct hotspots of cell response resulted from treatment with specific combinations of factors (Fig. 2D).

### FGF‐2 and HS8 Individually and Additively Promote hMSC Proliferation and Expansion

**Figure 3:**
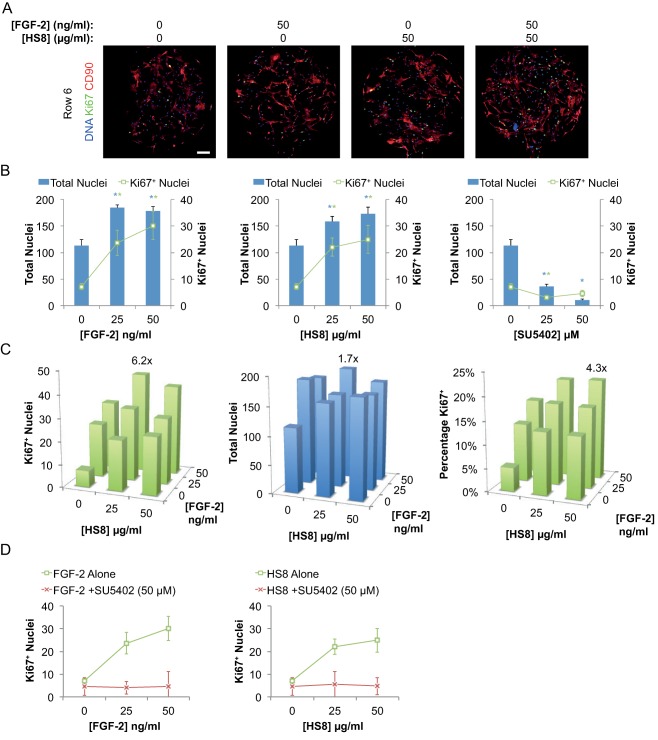
Analysis of microbioreactor arrays (MBA) screen. **(A)**: Fluorescence microscopy images of individual chambers (∼1.63 mm diameter) from Row 6 in MBA (corresponding to data in Fig. 2D), with treatment conditions as shown. Scale bar, 200 µm. **(B)**: Plots from selected individual MBA columns showing effects of titrations of individual factors (FGF‐2, HS8, and SU5402) on total and Ki67^+^ nuclei. Bars represent mean of all 10 chambers in the relevant column ± SEM. Blue * and green * indicates *p* < .05 versus control (zero concentration; Column 1, Fig. 2D) for total nuclei (blue) and Ki67^+^ nuclei (green) by ANOVA, Tukey post hoc test. **(C)**: Plots showing effects of combinations of FGF‐2 and HS8 in absence of SU5402, on Ki67^+^ nuclei, total nuclei and percentage Ki67^+^ nuclei. Bars represent mean of all 10 chambers in the relevant column. Maximum responses are annotated with fold of control (Column 1, Fig. 2D). **(D)**: Plots of Ki67^+^ nuclei for titrations of FGF‐2 and HS8 in the absence and presence of SU5402 at 50 µM. Points represent mean of all 10 chambers in the relevant column ± SEM. Abbreviations: FGF‐2, fibroblast growth factor‐2; HS8, heparan sulfate variant.

Examining images of individual chambers, it was evident that addition of either FGF‐2 (50 ng/ml) or HS8 (50 µg/ml) resulted in a better coverage of CD90^+^ hMSCs across the chamber's culture surface, compared to nonsupplemented conditions. These factors also resulted in increased numbers of both total cells and Ki67^+^ proliferating cells, and the effect was even more apparent when the two factors were combined together (Fig. 3A).

Quantifying the effects of individual factors by taking average measurements from individual MBA columns (as in Fig. 2D), it was clear that addition of either FGF‐2 or HS8 resulted in dose‐dependent increases in both Ki67^+^ nuclei and total nuclei, suggesting induction of hMSC proliferation and expansion (Fig. 3B). FGF‐2 at 50 ng/ml (Column 19, Fig. 2D) caused increased Ki67+ nuclei to 4.2‐fold and total nuclei to 1.6‐fold of control conditions (Column 1, Fig. 2D). HS8 at 50 µg/ml (Column 7, Fig. 2D) had a comparable effect, increasing Ki67+ nuclei to 3.5‐fold and total nuclei to 1.5‐fold of control. These increases were statistically significant (*p* < .05 by ANOVA, Tukey post hoc test) when comparing variation of the 10 culture chambers in the column (Fig. 3B). Cells exposed to SU5402, on the other hand, failed to undergo proliferative expansion (Fig. 3B).

For conditions where both FGF‐2 and HS8 were present in varying ratios, the two factors in combination were able to further enhance cell proliferation and cell numbers (Fig. 3C). The maximal response relative to control conditions (Column 1, Fig. 2D) was 6.2‐fold in terms of Ki67^+^ proliferating cells and 1.7‐fold in terms of total nuclei, which occurred in conditions of 50 ng/ml FGF‐2 and 25 µg/ml HS8 (Column 22, Figs. 2D, 3C). However, the maximum percentage of Ki67^+^ proliferating cells occurred at the maximum levels of the two factors (50 ng/ml FGF‐2 and 50 µg/ml HS8; Column 25, Figs. 2D, 3C). The factors appeared to have an additive rather than synergistic effect, with a possible rate‐limiting or saturation mechanism at higher doses. This suggests their effect on proliferation may act through a common mechanism, presumably FGF‐2‐driven signaling through an HS‐FGFR1 complex. This is supported by the effect of the FGFR1 tyrosine kinase inhibitor SU5402 in blocking the enhancing effects of HS8 or FGF‐2 supplementation (Figs. 2D, 3D). We did not expect the plateauing of response to result from contact inhibition, as cultures could be continued until ∼6 days with continued cell expansion up to ∼600 nuclei per chamber (data not shown).

### FGF‐2 and HS8 Alter the Paracrine‐Dependent Response Profile of hMSCs

**Figure 4:**
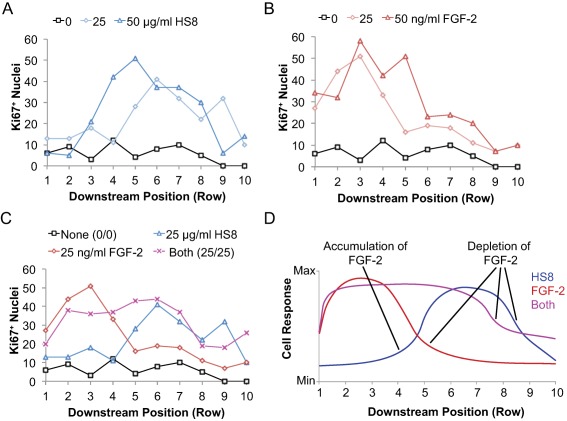
Paracrine profiles of cell responses. **(A–C)**: Plots of Ki67^+^ nuclei in each chamber versus downstream position (Row coordinate of the chamber) for (A) increasing concentrations of HS8, (B) increasing concentrations of FGF‐2, and (C) combinations of HS8 and FGF‐2. Data from Row 9 and 10 for the control condition (0/0) were flagged for quality control and are represented as zero. **(D)**: Conceptual model of the paracrine‐dependent effects on cell responses based on data in (C). Abbreviations: FGF‐2, fibroblast growth factor‐2; HS8, heparan sulfate variant.

Medium in the MBA continuously flows downstream through a column of 10 serial culture chambers and is then discarded. We observed that numbers of Ki67^+^ and total nuclei varied considerably from Row 1 to 10 down the columns in the MBA. Tracing the responses along from the first chamber (Row 1) to last chamber (Row 10) in a column, the changes generally presented with smooth gradients (Fig. 2D), rather than stochastically arranged high and low responses. Since the continuously flowing medium is subject to progressive depletion of exogenous factors and accumulation of freely diffusible endogenous factors, a changing response profile from upstream to downstream chambers (Rows 1 to 10) is a signature we have previously associated with varying levels of diffusible factors in the medium in each of the serial chambers. HS8 when added alone at 25 µg/ml caused an elevated number of Ki67^+^ cells, but importantly this was predominantly in the downstream region of the column (Rows 5 to 9) (Fig. 4A). When the amount of HS8 added was increased to 50 µg/ml, this peak shifted upstream to Rows 3 to 8 (Fig. 4A). Conversely, irrespective of dose, FGF‐2 supplementation alone caused an increase in Ki67^+^ proliferating cells predominantly in the upstream half of the column (Rows 1 to 5) (Fig. 4B). Adding HS8 together with FGF‐2 caused a proliferative effect at levels comparable or above that of FGF‐2 alone, and this was sustained through all rows (Fig. 4C). Modeling the effect of paracrine factors on cell responses provides useful insights into the way HS8 and FGF‐2 might interact to alter the distribution of their responses (Fig. 4D).

### Replication of Factor Effects in Multiple hMSC Donors

To statistically confirm the effects of HS8 and FGF‐2 on hMSC proliferation and expansion, MBA runs were repeated and also completed with additional hMSC donors. Human MSCs (Donors B and C) were derived from bone marrow mononuclear cells by plastic adherence selection and culture, and assayed in a manner similar to Figure 2 (Donor B, Supporting Information Fig. 5; Donor C, Supporting Information Fig. 6). Baseline proliferation in these donors used at passage 3 was higher and consequently they were screened for 2 days (Supporting Information Figs. 5A, 6A). Similar to Donor A, proliferation and cell number expansion in Donors B and C were dose‐dependently stimulated by both FGF‐2 and HS8, and inhibited by SU5402 (Supporting Information Figs. 5B, 5C, 6B, 6C). Like Donor A, the expression of Ki67^+^ was dependent on the downstream position and the response profile was modulated with various treatments of FGF‐2 and HS8 (Supporting Information Figs. 5B, 6B). Overall, the responses of Donors B and C reflected most features of Donor A, providing confidence that factor effects apply to hMSCs generally. However, donor‐to‐donor variability in factor responses was also measured, particularly prominent in Donor C, for which factor sensitivity seemed weaker (Supporting Information Fig. 6).

### Factorial Analysis of Multiple hMSC Donors in Multiple MBA Runs

**Figure 5:**
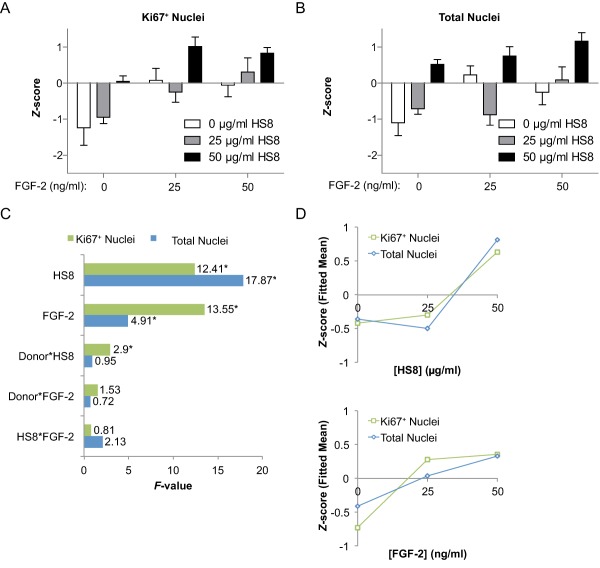
Replication and factorial analysis of MBA screen. **(A, B)**: Normalized cell responses for combinations of FGF‐2 and HS8 on Ki67^+^ nuclei (A) and total nuclei (B). *Z*‐score represents global standard deviations away from global mean for each run. Bars represent mean ± SEM of *Z*‐score for *n* = 6 runs (data from two MBA runs each from three donors A, B and C; one outlier is removed from one run from Donor B, with *p* < .05 by Grubbs' outlier test 33). **(C)**: Factorial analysis of single and combined factor effects, from data as in panels A and B. *F*‐value measuring relative significance of effect is plotted for each of the single factors FGF‐2 and HS8, as well as the the combined factors and interactions with Donor, for Ki67^+^ nuclei and total nuclei. * indicates *p* < .05. **(D)**: Fitted means from a reduced factorial model with insignificant (*p* > .05) terms removed, showing individual factor effects for FGF‐2 and HS8 on Ki67^+^ nuclei and total nuclei. Abbreviations: FGF‐2, fibroblast growth factor‐2; HS8, heparan sulfate variant.

Next, in order to estimate the significance of factor effects across multiple experimental runs and hMSC donors, column mean data from FGF‐2‐ and HS8‐containing conditions (omitting SU5402 data) from 2 runs from each of the 3 donors A, B, and C were standard normalized and pooled. These data showed the progressively additive effects of FGF‐2 and HS8, until the maximum average responses across the multiple donors and runs were obtained with 50 ng/ml FGF‐2 and 50 μg/ml HS8 (Fig. 5A, 5B). Factorial analysis of these data was then performed to identify the individual or combinations of factors with significant effects on the cell responses, Ki67^+^ nuclei and total nuclei. Plotting the *F‐*value from factorial analysis, which measures the relative significance of a factor's effect, showed that significant (*p* < .05) factor effects were clearly apparent for FGF‐2 and HS8 (Fig. 5C), with these two factors having the strongest significance. There was no synergistic effect (interaction effect) for FGF‐2 and HS8 combined (FGF‐2*HS8), reaffirming the additive nature of their interaction. However, there was an interaction effect on Ki67^+^ nuclei identified for Donor with HS8 (Donor*HS8), meaning there was some donor dependence on the proliferative response to HS8 (Fig. 5C). This is unsurprising, as HS8's effect seems to leverage some donor‐inherent characteristics such as endogenous FGF‐2 production rates. When the factorial model was reanalyzed with insignificant (*p* > .05) terms removed by backward elimination, FGF‐2 and HS8 were the only terms left in the model, as well as Donor*HS8, for Ki67^+^ Nuclei. Plots of fitted means for these individual factor effects for FGF‐2 and HS8 on Ki67^+^ nuclei and total nuclei all showed positive increasing relationships (Fig. 5D), summarizing their effects on hMSC proliferation and expansion when measured by 6 runs in 3 hMSC donors.

### HS8 Modulates FGF‐2 Partitioning Between Cell Surface and Supernatant

**Figure 6:**
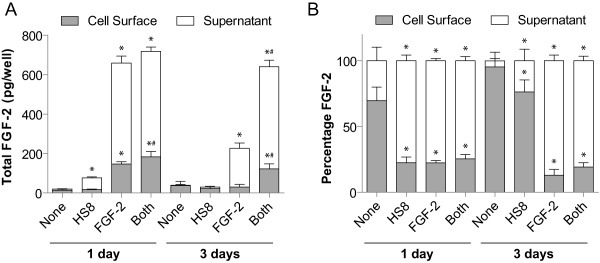
Measurement of FGF‐2 Levels on Cell Surface and in Supernatant by enzyme‐linked immuno‐sorbent assay. **(A)**: FGF‐2 levels on the cell surface and in the supernatant for hMSCs grown for 1 day and 3 days in control medium (None), or with addition of 25 μg/ml HS8 (HS8), 25 ng/ml FGF‐2 (FGF‐2), or both HS8 and FGF‐2 (Both). Bars represent mean ± SD for *n* = 6 measurements (pool of triplicate cultures from both of Donors A and B). * indicates *p* < .05 against control (None) condition from the same time point (two‐way ANOVA with Bonferroni post hoc tests); # indicates *p* < .05 for the Both condition against the FGF‐2 condition from the same time point. **(B)**: Percentage distribution of total FGF‐2 between cell surface and supernatant compartments from data in (A). Abbreviations: FGF‐2, fibroblast growth factor‐2; HS8, heparan sulfate variant.

Based on data from the MBA, we next tested whether the presence of HS8 altered the levels and partitioning of FGF‐2 molecules between the cell surface and the medium supernatant in standard static cultures. Human MSC donors (Donors A and B) were grown for 1 or 3 days in the presence of control medium, and media containing HS8, FGF‐2, or both HS8 and FGF‐2. Then, FGF‐2 levels in the supernatant medium and the cell surface fraction (isolated with a 2 M NaCl wash) were measured by ELISA (Fig. 6). These measurements revealed that in control medium (None), small amounts of FGF‐2 (38 pg/well) were detected on the cell surface over a 3‐day period (Fig. 6A), with the majority of the FGF‐2 (95%) located on the cell surface (Fig. 6B). On addition of HS8 however, a significant increase in the level of FGF‐2 was detected in the supernatant after 1 day (11.8‐fold of control, *p* < .001), returning to baseline levels after 3 days (Fig. 6A). In parallel, levels of cell surface FGF‐2 are maintained, yet an increased total amount of FGF‐2 is detected after 1 day. Since we measured FGF‐2 levels in the FBS‐containing background medium of only ∼5.5 pg/ml (∼2.2 pg/well), the increase in FGF‐2 in the supernatant that was detected after 1 day treatment with HS8 alone (∼53 pg/well) is likely to result from increased production, release and stabilization of endogenous FGF‐2 rather than from FGF‐2 present in the initial FBS‐containing medium, since no other FGF‐2 was added to this condition. This suggests HS8 increases endogenous FGF‐2 production, increases FGF‐2 transport into bulk supernatant, and stabilizes FGF‐2 within the supernatant. The partitioning of increased amounts Fig. 6A) and proportions (Fig. 6B) of FGF‐2 into the supernatant suggests HS8 competes with endogenous HS for FGF‐2 binding, releasing FGF‐2 from the cell surface and making it available in the supernatant. In the MBA, we use the proxy of cell growth, which is critically dependent on FGF‐2, to readout the activity of FGF‐2, contributed in exogenous and endogenous manners. However, in the ELISA measurements of static cultures, we measured FGF‐2 levels with direct comparison between conditions with the presence and absence of exogenously added FGF‐2. This allowed us to make a good assessment of endogenous and exogenous factor contribution.

Next, in conditions where exogenous FGF‐2 was supplemented, FGF‐2 could be detected at highly elevated levels both on the cell surface and in the supernatant after 1 day, relative to control cultures. By 3 days, however, the cell surface FGF‐2 had returned to baseline levels (*p* > .05 vs. control) and the FGF‐2 in the supernatant decreased (197 pg/well, *p* < .001) from the level at 1 day (512 pg/well). However, adding HS8 together with FGF‐2 (Both) over a 3‐day period elevated FGF‐2 levels were detected relative to FGF‐2 alone, both on the cell surface (4.1‐fold of FGF‐2 condition, *p* < .001) and in the supernatant (2.6‐fold of FGF‐2 condition, *p* < .001). It should be noted these data were not normalized to cell numbers, which could account for some differences in total FGF‐2 levels, however the maximum increase in cell numbers observed in the MBA experiments was only to 1.7‐fold of control conditions. Together, these data suggest a dual role for HS8 acting on endogenous and exogenous FGF‐2. HS8 increases the levels and availability of endogenous FGF‐2, and also stabilizes exogenous FGF‐2 over extended periods. Both these mechanisms contribute to a greater integrated amount of FGF‐2 being available to the culture over time.

## Discussion

Ex vivo hMSC expansion to clinically significant numbers whilst maintaining therapeutic potency remains a challenge. Critical growth factors for hMSCs (for example FGF‐2, and PDGF‐BB 34, 35) have been identified as being endogenously produced by the cells themselves 8, 12, 36, but the impact of these endogenous factors on hMSC expansion and properties is relatively poorly understood. This is partly because of a shortage of methods to effectively manipulate and evaluate the cell endogenous factors, but microfluidic systems are providing new methods for working with them 37. Endogenous factors might therefore represent untapped resources with beneficial impacts on hMSC expansion ex vivo that could be exploited improve the output and cost‐effectiveness of hMSC expansion during clinical scale‐up. These factors are known to be sequestered by HS chains on cell surface HSPGs, which control their deployment and activity in physiological processes 23, 38, 39, 40, 41 such as development, morphogenesis, and homeostasis. In this work, we focused on the role of FGF‐2 as a key factor driving hMSC expansion, as well as its transport within hMSC cultures, particularly its crucial interactions with its coreceptor HS. In our experiments, conditions that caused maximal hMSC proliferation and expansion (i.e., when both FGF‐2 and HS8 were present) also had the highest sustained levels of FGF‐2 available on the cell surface and in the supernatant (Fig. 6). This correlation might underscore FGF‐2 availability as being a bottleneck in hMSC expansion, which lies upstream of the rate‐limiting step for hMSC expansion identified previously: FGFR1 signaling activity 11.

Using a microbioreactor array (MBA) that permits combinatorial assessment of exogenous and paracrine factors, we surveyed hMSC responses to such factor combinations. This improved strategy allowed us to decipher combinatorial effects of ligands like FGF‐2 and the affinity‐purified GAG HS8 on hMSC growth by monitoring direct and paracrine‐dependent cell responses. This approach provides unique insight because the MBA simultaneously provides combinatorial mixes of factors to hMSCs under continuous flow, which allows for the influence and identity of paracrine factors to be probed, using cell readouts along with blocking antibodies, signaling pathway inhibitors, or conditioned medium 5. MBA screening resulted in the most critical insight of this work: an understanding of the paracrine‐dependent modulation of hMSC growth by HS8. These were augmented with static culture measurements of FGF‐2 levels to better understand HS8's effects on FGF‐2 transport in hMSC cultures.

In the MBA, continual flow of medium is provided in a 100 μm‐high channel directly over the cell layer. Since cells in the first row (chamber) of a column of 10 chambers see fresh medium, and cells in subsequent rows see progressively more depleted and conditioned medium, the row coordinate acts as a surrogate measure of the degree of depletion (removal of nutrients and exogenous factors) and conditioning (accumulation of wastes and secreted endogenous factors) of the medium. Because nutrient provision and waste accumulation are controlled (within a viable range for cell growth) by matching the medium exchange rate to time‐averaged static conditions, we expect most of the variation down a column to be caused by differing levels of exogenous and diffusible paracrine factors. Plotting an appropriate cell response against the row coordinate can therefore provide insight into its dependence on these levels. Using this innovative approach, we uncovered paracrine‐dependent modulation of hMSC proliferative responses, which cannot be easily identified in standard static culture systems. However, the detection of paracrine‐dependent responses allowed us to generate hypotheses that could then be verified in static cultures.

**Figure 7:**
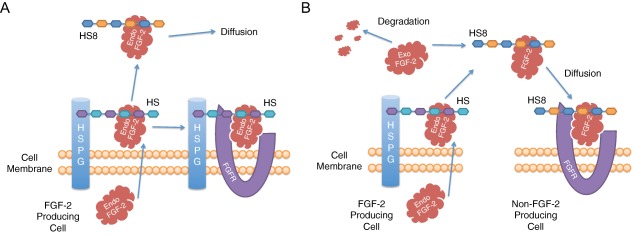
Conceptual diagram of FGF‐2 transport. **(A)**: Endogenous FGF‐2 export requires HS proximal to the cell surface, which then promotes autocrine association with FGFR. Diffusible HS8 liberates endogenous FGF‐2 from the cell surface by competition with HSPG‐bound cell surface HS. HS8‐bound FGF‐2 is then free to diffuse to other cells. **(B)**: Exogenous FGF‐2 degrades over time, but can be bound and stabilized by HS8. The HS8‐bound FGF‐2 pool is then free for diffusion to other cells, including those that do not produce FGF‐2 or display appropriate cell surface HS. Abbreviations: Endo FGF‐2, endogenous FGF‐2; Exo FGF‐2, exogenous FGF‐2; FGF‐2, fibroblast growth factor‐2; HS8, heparan sulfate variant; HS, endogenous cell surface heparan sulfate; HSPG, heparan sulfate proteoglycan; FGFR, FGF receptor.

Because endogenous, cell surface HS binds endogenous FGF‐2 on export and either promotes autocrine association with FGFR or facilitates juxtacrine transfer between touching cells 18, we reasoned that the freely‐diffusible HS8 competes with this cell surface HS to bind the endogenous FGF‐2, releasing it from the cell surface, stabilizing it, and allowing transfer to other cells by paracrine diffusion (conceptualized in Fig. 7A). This is supported by our observation that cells treated with only HS8 had more endogenous FGF‐2 available in the supernatant after 1 day (Fig. 6A), and the observation that cells treated with only HS8, under continuous flow in the MBA, proliferated primarily in downstream chambers (Fig. 4A). This suggests a requirement for endogenous factors (i.e., FGF‐2) to be released from upstream cells and progressively accumulate until reaching levels sufficient to drive robust cell responses. Given that SU5402 (FGFR antagonist) blocked the ability of HS8 to stimulate cell proliferation in downstream chambers (Figs. 2D, 3D), our data supports endogenous FGF‐2 being released from the cell surface by binding to HS8, and then acting on downstream chambers to stimulate proliferative responses.

Exogenous FGF‐2 normally undergoes degradation (primarily thermal aggregation 19), but can be stabilized by binding HS8, making higher levels of it available in the supernatant. Free HS8/FGF‐2 complexes in the supernatant can then diffuse to other cells including those which may not produce FGF‐2 or display appropriate endogenous cell surface HS, increasing overall FGF‐2 stimulation of the cell population (conceptualized in Fig. 7B). This is supported by our measurements of higher levels of FGF‐2 both in the supernatant and on the cell surface when HS8 was supplemented along with FGF‐2, compared to FGF‐2 alone, particularly the fact that is was sustained through 3 days (Fig. 6A). Previous work has shown that HS and heparin can prevent depletion of exogenous FGF‐2 from the supernatant by cell surface HS and increase its radius of diffusion over a monolayer of cells 21. In our work, adding FGF‐2 alone induced a burst of proliferation that occurred in the upstream culture chambers and then diminished moving to downstream chambers (Fig. 4B), suggesting that FGF‐2 is readily bound by cells in the upstream chambers and thereby depleted from the medium, making less available to the downstream chambers. As well as this, some thermal degradation should occur during the transit of FGF‐2 through the column (∼1.5 hours average residence time). However, when HS8 was added to FGF‐2, a heightened proliferative response was sustained down the entire column of chambers (Fig. 4C). This highlights the ability of HS8 to prevent immediate depletion of the FGF‐2 pool by cell surface HS through competitive binding, and to extend the effective range of FGF‐2 stimulation by stabilizing it within the supernatant.

The major implications of this work for hMSC scale‐up are that an engineered carbohydrate adjuvant (HS8) can cause disruption of native growth factor‐HSPG feedback loops to alter the distribution of FGF‐2 molecules, thereby enhancing hMSC expansion. Importantly, using an MBA screening strategy, we were able to determine that several regulated systems are modulated by HS8: levels of growth factor production, stability and transport mechanisms. This results in an overall increased level of growth factor cycling through the critical FGF‐HS‐FGFR system, and consequently gains in expansion of the hMSC population.

## Conclusion

HS8 was shown to act additively with FGF‐2 for enhanced ex vivo expansion of hMSCs. Microbioreactor array experiments and measurements of FGF‐2 levels in static cultures suggest HS8 increases the overall amount and duration of FGF‐2 available to the whole population of hMSCs in culture. This occurs by increasing production and facilitating diffusion of endogenous FGF‐2, and stabilizing exogenous FGF‐2 within the supernatant. Supplementation of hMSC cultures with HS8 could therefore be an effective strategy to enhance hMSC expansion, especially in large‐scale bioreactor systems.

## Author Contributions

D.M.T: conception and design, collection and/or assembly of data, data analysis and interpretation, manuscript writing, final approval of manuscript; C.T.: collection and/or assembly of data, manuscript writing, final approval of manuscript; N.G.: provision of study material or patients, final approval of manuscript; V.N.: conception and design, financial support, final approval of manuscript; J.C.‐W.: financial support, provision of study material or patients, final approval of manuscript; S.C.: conception and design, financial support, provision of study material or patients, data analysis and interpretation, manuscript writing, final approval of manuscript.

## Disclosure of Potential Conflicts of Interest

The authors indicate no potential conflicts of interest.

## Supporting information

Supporting Information.
