# Supplementary material for: Microfluidic Screening Reveals Heparan Sulfate Enhances Human Mesenchymal Stem Cell Growth by Modulating Fibroblast Growth Factor‐2 Transport
Source: Stem Cells Transl Med. 2017 Feb 16;6(4):1178–90. doi: 10.1002/sctm.16-0343 (PMC5442852; doi:10.1002/sctm.16-0343)
Supplement: Supplementary file 1 — Supporting Information. [file SCT3-6-1178-s001.pdf]

Microfluidic Screening Reveals Heparan Sulfate Enhances Human Mesenchymal Stem Cell Growth by Modulating FGF-2 Transport

Supplementary Information

Drew M. Titmarsh[1], Clarissa L.L. Tan[1], Nick R. Glass[2], Victor Nurcombe[1,3], Justin J. Cooper-White[2,4,5], and Simon M. Cool[1,6*]

[1]Institute of Medical Biology, Agency for Science Technology and Research (A*STAR), 8A Biomedical Grove, #06-06 Immunos, SINGAPORE 138648.

[2]Australian Institute for Bioengineering & Nanotechnology, The University of Queensland, St. Lucia, QLD 4072, AUSTRALIA.

[3]Lee Kong Chian School of Medicine, Nanyang Technological University–Imperial College London, SINGAPORE 308232.

[4]School of Chemical Engineering, The University of Queensland, St. Lucia, QLD 4072, AUSTRALIA.

[5]Biomedical Manufacturing, Manufacturing Flagship, CSIRO, Clayton, Victoria 3169, AUSTRALIA.

[6]Department of Orthopaedic Surgery, Yong Loo Lin School of Medicine, National University of Singapore, SINGAPORE 119074.

*Corresponding author:
Simon Cool, PhD
Institute of Medical Biology
8A Biomedical Grove, #06-06 Immunos, SINGAPORE 138648
Tel +65-64070176 Fax: +65-64789477
Email: simon.cool@imb.a-star.edu.sg

SUPPLEMENTARY FIGURES

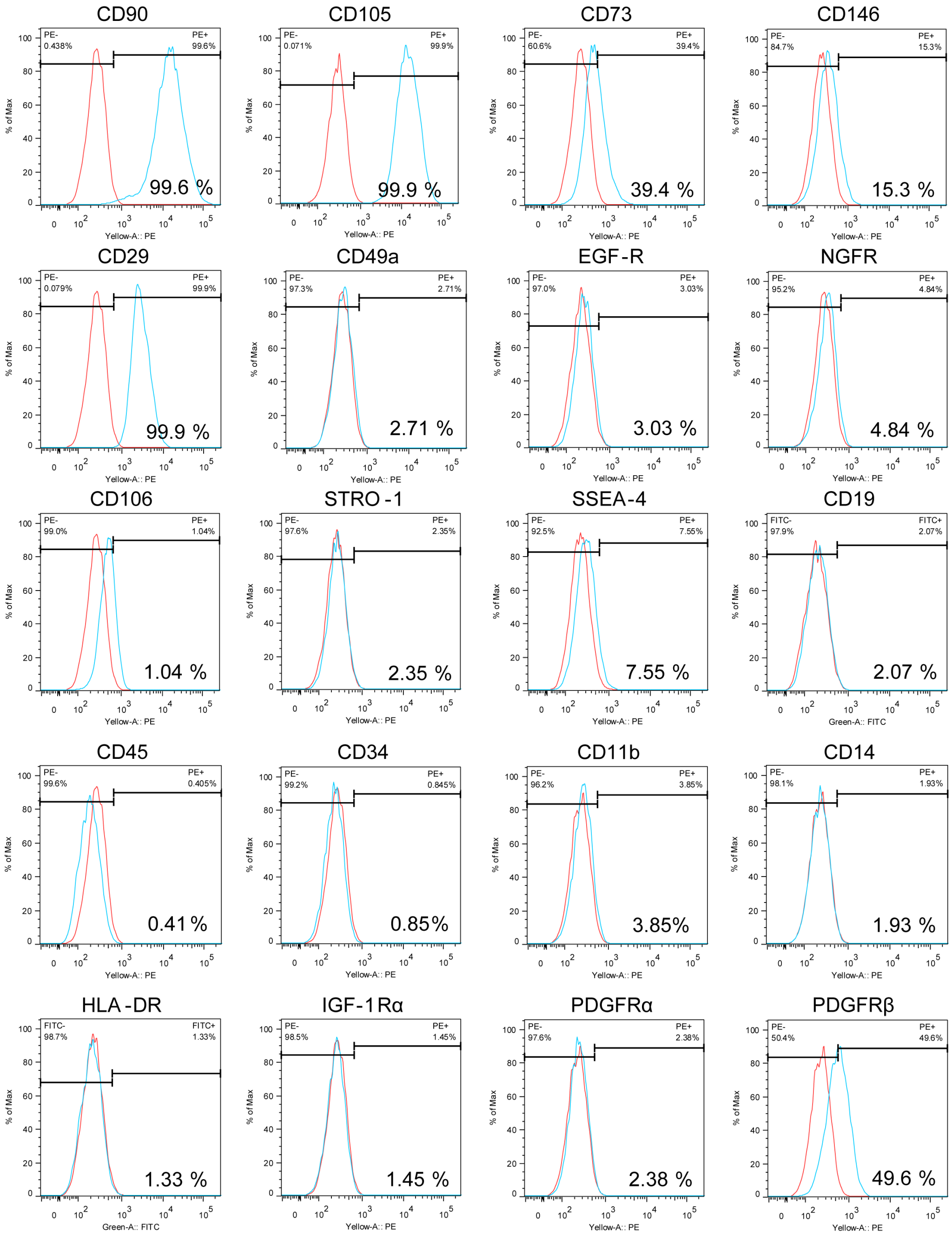

Supplementary Figure 1 – Flow Cytometric Immunophenotyping of hMSCs derived from Donor B. Cells were fixed at passage 6 and analysed by flow cytometry for a range of markers including hMSC markers CD90, CD105 and CD73, hematopoietic markers CD45, CD34, CD11b, CD14 and HLA-DR. Percentage positive cells are marked. The cutoff was set for a false-positive rate of < 2%, based on the relevant isotype control.

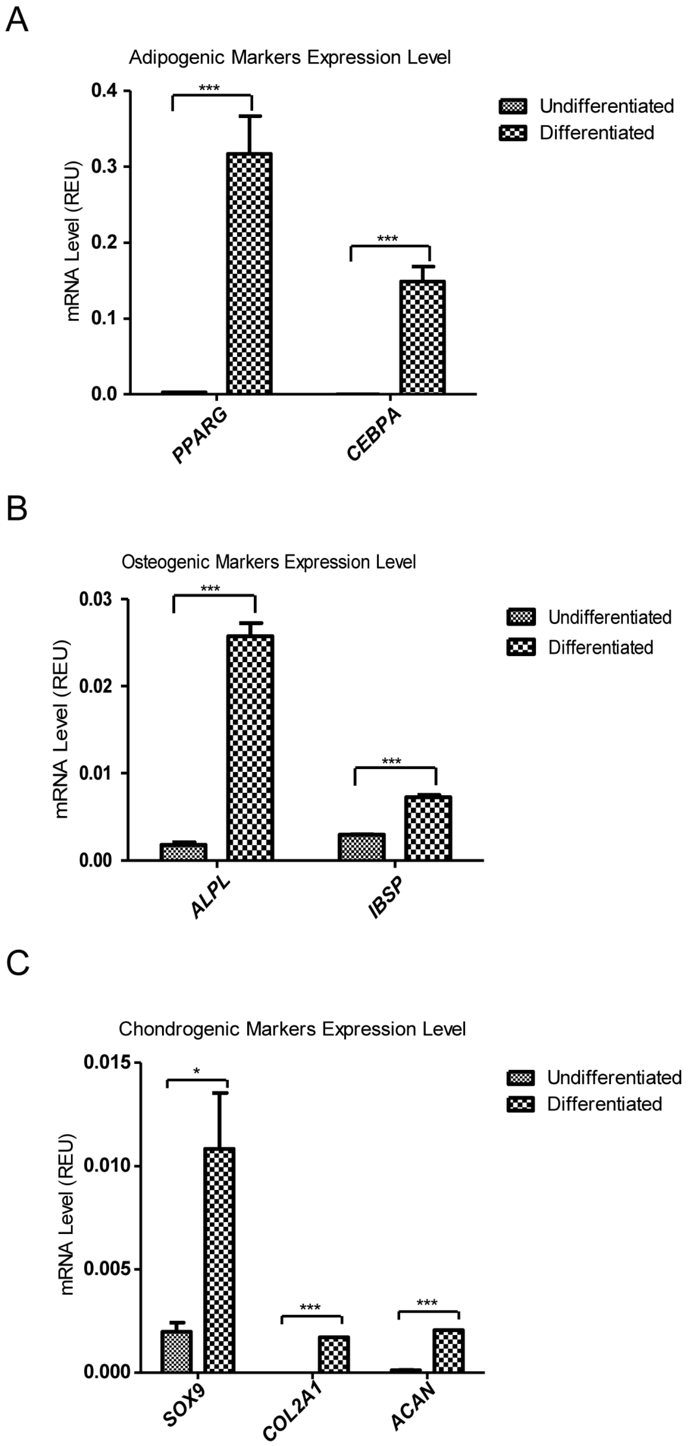

Supplementary Figure 2 – RT-qPCR Quantification of Expression of Multi-lineage Differentiation Markers by hMSCs derived from Donor B. A Adipogenic markers, **B** osteogenic markers, and **C** chondrogenic markers were evaluated from RNA taken from undifferentiated and differentiated cells from Donor B. Columns represent mean mRNA level in relative expression units to reference gene *ACTB*, +/- S.D. of triplicate cultures, * - $p < 0.05$ and *** - $p < 0.001$ by unpaired Student's *t*-test for each gene.

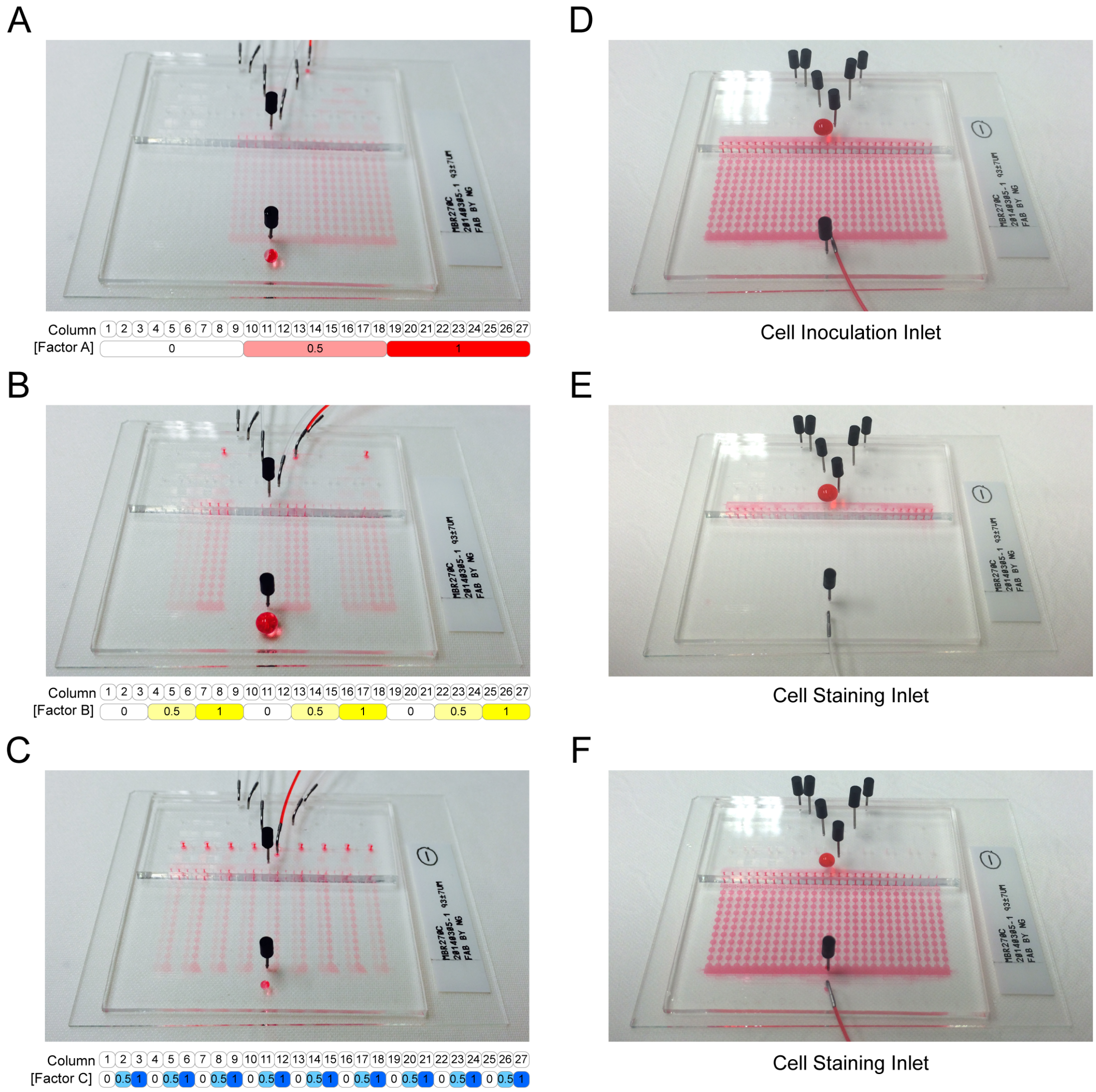

Supplementary Figure 3 - MBA Dye Loading Validation. A-C Photographs of dye loading of Ponceau S solution through individual factor inlets A, B, and C (**A**, **B** and **C**, respectively). The panel below shows the design concentration levels of factor in each column of the array. **D** Dye loading through the cell inoculation inlet, with factor/buffer inlets plugged closed. **E,F** Dye washout (**E**) and re-loading (**F**) from the cell staining inlet, with factor/buffer inlets plugged closed.

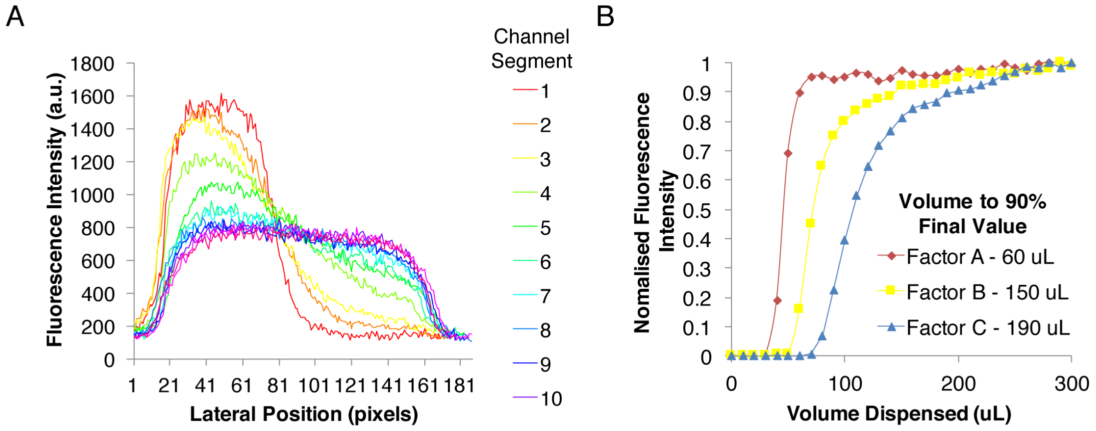

Supplementary Figure 4 – Dynamic Filling and Diffusive Mixing of Alexa Fluor 488-labelled Heparin in the MBA. A Progression of diffusive mixing in the MBA. Pixel intensity profiles were measured across successive microchannel segments during diffusive mixing. Pixel intensity was measured across the channel width in successive segments. **B** Dynamic tracking of establishment of the maximum concentration levels in each of the MBA factor channels A, B and C is shown. Evolution of fluorescence was tracked starting from the beginning of syringe pump flow, for 1 hour at 300 μL/h total flowrate. Volume to 90% final value is shown for each factor channel. All factor channels reached the steady state value within 300 μL volume delivery.

A

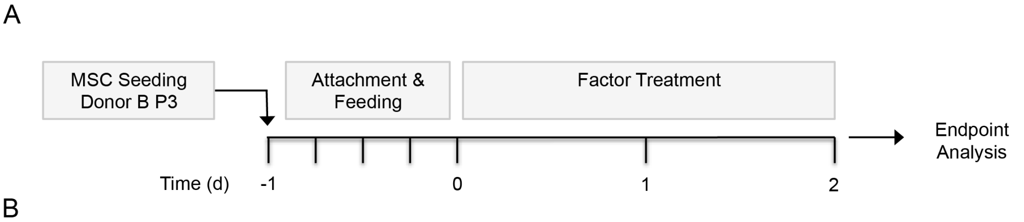

B

Column	1	2	3	4	5	6	7	8	9	10	11	12	13	14	15	16	17	18	19	20	21	22	23	24	25	26	27	Carrier
[HS8]	0									25									50									+0.1% BSA
[FGF-2]	0			25			50			0			25			50			0			25			50			+0.1% BSA
[SU5402]	0	25	50	0	25	50	0	25	50	0	25	50	0	25	50	0	25	50	0	25	50	0	25	50	0	25	50	+30% FBS

Final Background Medium: DMEM-LG +10% FBS +4 mM L-Gln +1% Pen/Strep +0.067% BSA

Ki67⁺ Nuclei

Row \ Column	1	2	3	4	5	6	7	8	9	10	11	12	13	14	15	16	17	18	19	20	21	22	23	24	25	26	27	Scale
1	97	57	11	186	76	7	162	79	6	140	75	8	131	55	9	135	95	18	172	63	11	169	73	7	169	68	9	220
2	92	52	6	203	54	8	212	91	6	133	51	3	169	69	2	134	87	20	210	85	3	178	93	6	152	67	19	193
3	105	42	6	195	66	11	197	66	2	138	54	8	154	49	10	202	65	23	220	54	6	148	48	8	163	66	11	165
4	54	23	10	214	76	8	212	67	4	104	47	7	130	58	7	138	116	18	193	64	6	175	64	8	174	46	9	138
5	91	38	8	212	73	9	202	52	4	89	40	2	148	41	6	135	85	19	153	62	13	187	61	5	211	86	13	111
6	56	48	6	162	46	5	148	36	8	119	28	3	98	34	3	96	83	7	182	76	4	212	92	7	186	66	5	83
7	45	25	7	174	56	5	130	40	5	60	22	6	169	52	4	101	77	7	169	79	4	182	94	13	200	58	3	56
8	67	31	6	160	49	2	182	34	4	94	24	5	145	68	11	136	91	13	144	56	13	168	71	22	143	94	13	28
9	68	28	2	166	53	2	167	36	7	65	23	3	199	77	12	184	95	6	142	63	4	169	75	6	171	110	5	1
10	44	43	1	166	45	2	123	20	4	70	23	3	182	73	11	167	106	17	185	58	2	144	51	8	180	60	3	QCF
Average	72	39	6.3	184	59	5.9	174	52	5	101	39	4.8	153	58	7.5	143	90	15	177	66	6.6	173	72	9	175	72	9	

Total Nuclei

Row \ Column	1	2	3	4	5	6	7	8	9	10	11	12	13	14	15	16	17	18	19	20	21	22	23	24	25	26	27	Scale
1	336	383	318	474	411	401	427	412	297	381	399	259	315	233	242	329	353	338	465	389	291	403	435	313	462	413	306	572
2	273	372	340	463	381	377	482	407	356	465	393	299	355	295	199	393	358	327	483	388	326	464	382	278	436	427	310	522
3	395	420	337	429	378	358	479	416	353	468	370	339	364	267	179	428	336	339	515	384	291	426	455	366	486	415	320	471
4	341	381	365	460	368	317	520	368	327	453	364	289	375	221	201	335	372	318	506	397	306	475	417	330	514	373	222	421
5	352	394	349	505	389	298	474	340	310	*	295	286	332	203	168	294	318	338	456	330	353	466	397	384	572	460	274	370
6	343	354	308	474	364	*	446	328	303	369	300	261	259	215	173	270	326	292	459	330	321	535	435	409	563	390	296	320
7	323	*	344	455	383	306	359	295	247	317	238	219	321	279	192	284	258	299	400	389	291	472	439	364	524	458	219	269
8	363	400	306	435	323	270	407	284	256	290	246	240	355	336	276	349	319	223	362	287	281	440	389	361	465	484	304	219
9	381	358	335	433	352	212	372	282	226	261	262	265	515	317	214	429	349	329	359	323	251	445	398	298	430	414	220	168
10	389	389	256	424	285	193	466	246	251	363	307	264	424	345	291	443	386	305	434	305	247	383	315	246	488	346	190	QCF
Average	350	383	326	455	363	304	443	338	293	374	317	272	362	271	214	355	338	311	444	352	296	451	406	335	494	418	266	

C

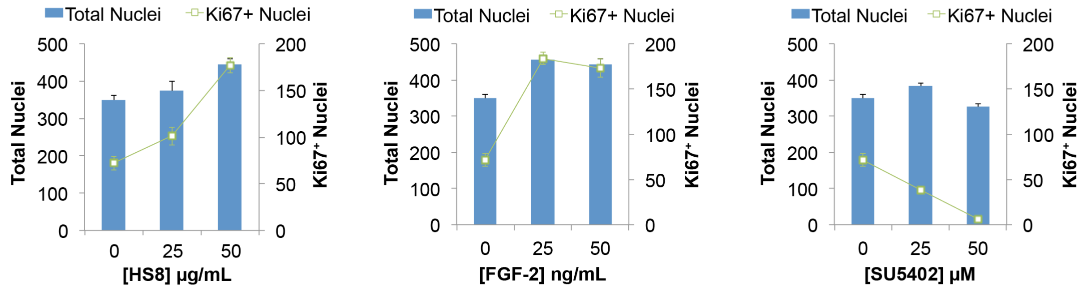

Supplementary Figure 5 - MBA Combinatorial Screening of HS8, FGF-2 and SU5402 in hMSC Donor B. **A** Experimental time course. **B** Top panel: compositions of media in each column of the MBA (HS8, µg/mL; FGF-2, ng/mL; SU5402, µM). Lower panels: Heatmaps of absolute numbers of Ki67⁺ nuclei, and total nuclei for each chamber in the MBA, corresponding to above compositions. Medium flow was from top (Row 1) to bottom (Row 10) down a column. Mean for each column is given below. QCF: data flagged for quality control issue during image processing. **C** Plots from selected individual MBA columns showing effects of titrations of individual factors (HS8, FGF-2 and SU5402) on total and Ki67⁺ nuclei. Bars represent mean of all 10 chambers in the relevant column ± SEM.

A

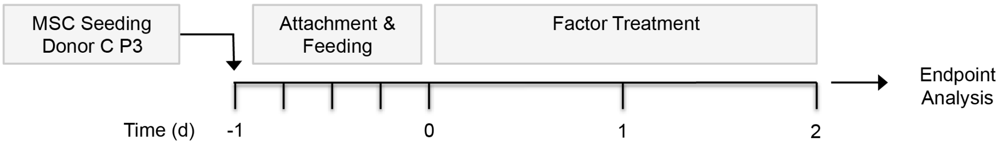

B

Column	1	2	3	4	5	6	7	8	9	10	11	12	13	14	15	16	17	18	19	20	21	22	23	24	25	26	27	Carrier
[HS8+]	0									25									50									+0.1% BSA
[FGF-2]	0			25			50			0			25			50			0			25			50			+0.1% BSA
[SU5402]	0	25	50	0	25	50	0	25	50	0	25	50	0	25	50	0	25	50	0	25	50	0	25	50	0	25	50	+30% FBS

Final Background Medium: DMEM-LG +10% FBS +1% L-Gln +1% Pen/Strep +0.067% BSA

Total Ki67+ Nuclei

Row \ Column	1	2	3	4	5	6	7	8	9	10	11	12	13	14	15	16	17	18	19	20	21	22	23	24	25	26	27	Scale
1	14	9	8	36	9	7	15	4	2	9	*	4	31	6	8	41	9	5	10	15	5	31	21	6	28	*	9	68
2	21	12	11	34	9	11	25	9	*	3	5	3	34	9	12	50	23	6	8	7	4	32	15	16	32	5	*	60
3	13	6	3	36	5	4	18	4	6	30	24	10	26	10	2	29	34	9	18	8	4	59	9	5	40	3	16	51
4	24	16	5	19	10	9	20	12	6	19	9	7	22	8	13	28	14	7	10	5	10	68	6	2	50	7	10	43
5	*	10	5	19	4	5	37	8	6	8	31	14	33	8	1	35	3	12	29	8	7	39	14	10	42	8	6	35
6	29	2	5	14	19	13	24	5	2	6	9	7	17	24	10	41	2	12	27	2	6	39	21	8	37	9	6	26
7	34	4	2	19	5	13	16	3	1	7	20	11	19	*	14	8	7	5	27	2	10	38	*	6	22	*	20	18
8	12	21	14	*	19	11	15	10	6	7	7	4	18	18	18	28	16	4	44	12	8	52	8	4	4	9	8	9
9	14	3	*	23	*	5	7	8	6	4	9	9	28	15	12	12	8	6	33	3	1	31	12	22	21	20	2	1
10	16	4	4	35	4	16	*	12	6	14	12	2	19	5	1	31	5	6	29	15	5	40	16	17	17	2	5	QCF
Mean	20	8.7	6.3	26	9.3	9.4	20	7.5	4.6	11	14	7.1	25	11	9.1	30	12	7.2	24	7.7	6	43	14	9.6	29	7.9	9.1	
Max	34	21	14	36	19	16	37	12	6	30	31	14	34	24	18	50	34	12	44	15	10	68	21	22	50	20	20	

Total Nuclei

Row \ Column	1	2	3	4	5	6	7	8	9	10	11	12	13	14	15	16	17	18	19	20	21	22	23	24	25	26	27	Scale
1	111	94	75	190	130	76	107	80	46	91	61	31	119	110	56	145	79	74	158	74	58	159	89	28	143	128	38	205
2	90	76	58	124	114	44	105	73	*	97	64	45	125	92	54	155	105	46	147	92	43	170	77	69	126	101	73	183
3	134	83	53	139	78	34	102	90	52	113	55	35	98	88	55	109	98	57	133	74	40	191	100	51	170	137	67	160
4	104	96	40	125	85	36	95	49	54	118	45	29	108	120	39	139	85	49	160	61	55	198	114	44	180	120	39	138
5	114	69	53	119	70	35	120	66	47	109	92	53	128	136	55	157	113	84	163	85	60	185	105	57	175	116	69	116
6	139	96	55	112	74	66	101	59	44	81	84	52	91	115	82	158	86	54	177	79	43	205	113	42	136	120	51	93
7	124	104	77	94	100	33	116	58	44	131	75	36	118	156	116	131	100	80	160	101	49	180	80	62	144	93	42	71
8	113	128	62	108	148	45	117	77	50	94	45	26	117	131	47	168	157	88	178	87	61	175	79	42	150	91	82	48
9	138	112	115	170	122	37	74	71	50	89	77	57	134	116	62	132	113	89	174	83	45	152	85	55	118	102	58	26
10	95	81	44	134	76	62	97	91	74	93	68	30	137	99	53	179	126	76	175	108	47	154	95	59	126	67	47	QCF
Mean	116	94	63	132	100	47	103	71	51	102	67	39	118	116	62	147	106	70	163	84	50	177	94	51	147	108	57	
Max	139	128	115	190	148	76	120	91	74	131	92	57	137	156	116	179	157	89	178	108	61	205	114	69	180	137	82	

C

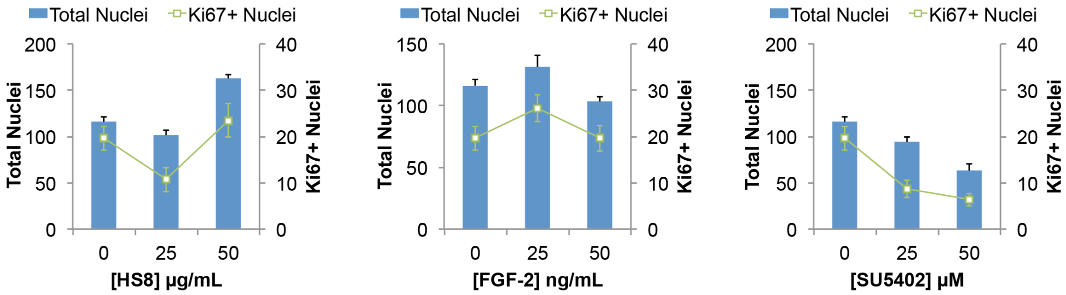

Supplementary Figure 6 - MBA Combinatorial Screening of HS8, FGF-2 and SU5402 in hMSC Donor C. **A** Experimental time course. **B** Top panel: compositions of media in each column of the MBA (HS8, μg/mL; FGF-2, ng/mL; SU5402, μM). Lower panels: Heatmaps of absolute numbers of Ki67+ nuclei, and total nuclei for each chamber in the MBA, corresponding to above compositions. Medium flow was from top (Row 1) to bottom (Row 10) down a column. Mean for each column is given below. QCF: data flagged for quality control issue during image processing. **C** Plots from selected individual MBA columns showing effects of titrations of individual factors (HS8, FGF-2 and SU5402) on total and Ki67+ nuclei. Bars represent mean of all 10 chambers in the relevant column ± SEM.

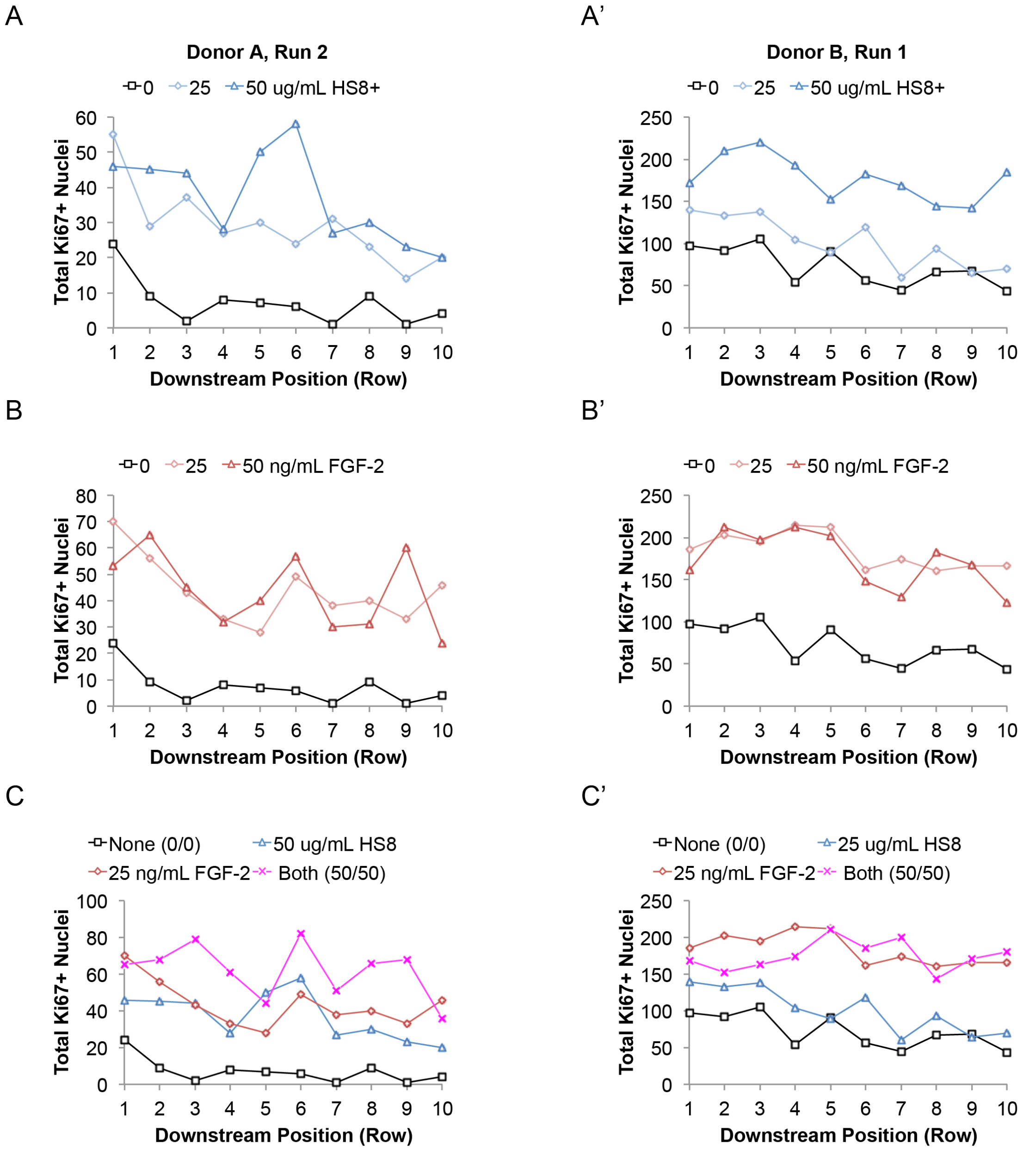

Supplementary Figure 7 – Paracrine profiles of cell responses in replicate runs and donors. A-C Plots for Donor A, Run 2, of Ki67⁺ nuclei in each chamber versus downstream position (Row coordinate of the chamber) for **A** increasing concentrations of HS8, **B** increasing concentrations of FGF-2, and **C** combinations of HS8 and FGF-2. **A'-C'** Corresponding plots for Donor B, Run 1.

SUPPLEMENTARY TABLES

Supplementary Table 1 – RT-qPCR Probe Information.

Gene Symbol	Gene Name	Assay ID	Reference Sequence
ALPL	alkaline phosphatase, liver/bone/kidney	Hs01029144_m1	NM_000478.4<br>NM_001127501.2<br>NM_001177520.1
IBSP	integrin-binding sialoprotein	Hs00173720_m1	NM_004967.3
PPARG	peroxisome proliferator-activated receptor gamma	Hs01115513_m1	NM_005037.5<br>NM_015869.4<br>NM_138711.3<br>NM_138712.3
CEBPA	CCAAT/enhancer binding protein (C/EBP), alpha	Hs00269972_s1	NM_004364.3
SOX9	SRY (sex determining region Y)-box 9	Hs01001343_g1	NM_000346.3
COL2A1	collagen, type II, alpha 1	Hs00264051_m1	NM_001844.4<br>NM_033150.2
ACAN	aggrecan	Hs00153936_m1	NM_001135.3<br>NM_013227.3
ACTB	actin, beta	Hs01060665_g1	NM_001101.3
